# The Role of Inositols in Endocrine and Neuroendocrine Tumors

**DOI:** 10.3390/biom14081004

**Published:** 2024-08-14

**Authors:** Marilda Mormando, Giulia Puliani, Marta Bianchini, Rosa Lauretta, Marialuisa Appetecchia

**Affiliations:** Oncological Endocrinology Unit, IRCCS Regina Elena National Cancer Institute, Via Elio Chianesi 53, 00144 Rome, Italy; marilda.mormando@ifo.it (M.M.); giulia.puliani@ifo.it (G.P.); marta.bianchini@ifo.it (M.B.); rosa.lauretta@ifo.it (R.L.)

**Keywords:** inositols, myo-inositols, inositol hexakisphosphate, endocrine cancer, thyroid cancer, neuroendocrine neoplasms

## Abstract

Inositols have demonstrated a role in cancer prevention and treatment in many kinds of neoplasms. Their molecular mechanisms vary from the regulation of survival and proliferative pathways to the modulation of immunity and oxidative stress. The dysregulation of many pathways and mechanisms regulated by inositols has been demonstrated in endocrine and neuroendocrine tumors but the role of inositol supplementation in this context has not been clarified. The aim of this review is to summarize the molecular basis of the possible role of inositols in endocrine and neuroendocrine tumors, proposing it as an adjuvant therapy.

## 1. Introduction

**Figure 1:**
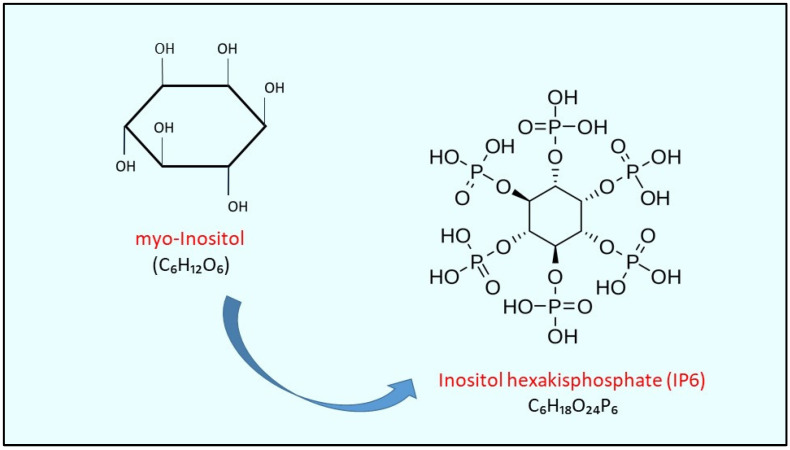
Myo-Inositol and its most phosphorylated form: inositol hexakisphosphate IP6.

The antiproliferative effect of myo-inositol (myo-Ins) represents, to date, an interesting object of study for researchers and oncologists. Myo-Ins is a cyclic polyalcohol with six carbon atoms and represents one of the nine stereoisomers of the inositol family. Its most phosphorylated form, inositol hexakisphosphate (IP6), is a highly represented component in vegetables, cereals and dried fruit (Figure 1). It is also contained in many mammalian cells and is involved in many cellular functions ranging from signal transduction to cell proliferation and differentiation [1]. For this reason, in recent years, studies focused on the prevention of tumors and the mechanisms of cell growth and metastasis of tumors have paid considerable attention to the role of inositols and IP6 [2]. Several studies focusing on nutrients such as polyphenolic acids, flavonoids and inositols have demonstrated their valuable and interesting anti-tumor effect, also reinforcing the idea of the Mediterranean diet, rich in vegetables and cereals, as a source of microelements essential for human health [3]. For some tumor types, such as colon, breast and prostate, diet seems to play a role in modifying their incidence [4]. It has been demonstrated that the consumption of fibers with a high content of IP6, the main myo-Ins supplier from foods, is able to reduce the incidence of colon cancer [5,6]. 

Although strong evidence in the literature demonstrated a reduction in tumor growth (*in vitro*) in different tumor types, such as breast, prostate, colorectal cancer, melanoma and leukemia [7,8,9,10], the role of inositols in endocrine cancers has not been fully elucidated despite the very promising assumptions. Currently, endocrinologists and gynecologists show a huge clinical interest in inositol and its derivatives (especially D-chiro-inositol) since it can be used as an insulin sensitizer in the treatment of polycystic ovary syndrome (PCOS) and infertility.

The aim of this review is to collect the available evidence on the role of inositols in cancer prevention and in the treatment of endocrine and neuroendocrine neoplasms. 

## 2. Materials and Methods

We performed a search of published English articles on the Pubmed database with the following keywords: “inositol”, “myo-inositol”, “IP6”, “endocrine cancer”, “neuroendocrine neoplasm”, “thyroid cancer”, “pituitary tumor” and “adrenal tumor”. Only articles reporting data on the role of inositols in endocrine and neuroendocrine neoplasms were considered for this review. This review was conducted according to the SANRA scale for the quality assessment of narrative review articles [11].

## 3. Results

### 3.1. Molecular Mechanisms of the Role of Inositols in Cancer

IP6 appears to be involved in cell cycle control with both cytostatic and cytotoxic effects [12,13]. Both IP6 and myo-Ins have been demonstrated to increase p53 oncosuppressive activity [14] and significantly reduce the expression of P13K and the activation of AKT, which are molecular pathways involved in cell growth [15,16]. Additionally, inositol modulates the Wnt/β-catenin pathway through cytoskeletal remodeling and β-catenin redistribution, thus contributing to substantive downregulation of different inflammatory markers [17]. IP6 has been shown to play a role in inducing cell apoptosis in several types of cancer *in vitro* and *in vivo* [18] and in inhibiting vascular endothelial growth factor (VEGF) secretion from tumor cells, which influences neoangiogenesis [19]. Furthermore, IP6 plays a crucial antioxidant role by reducing carcinogenesis mediated by active oxygen species and decreasing cellular damage through antioxidant function [20]. InsP6 and inositol also modulate the immune system and immunosuppression in the tumor microenvironment, increasing killer cell activity *in vitro* and neutralizing the carcinogen-induced depression of natural killer cell activity *in vivo* [21,22]. A synergistic anticancer action of IP6 combined with inositol was observed in animal models of colon and mammary tumor and metastatic fibrosarcoma [23,24,25]. 

In the context of endocrine and neuroendocrine tumors, it is important to consider the role of insulin resistance in cancer development. Myo-Ins is a component of the cell membrane, where it can be found as phosphatidyl-myoinositol, the precursor of inositol triphosphate (IP3), which acts as a second messenger in the transduction of several endocrine signals, including FSH, TSH and insulin [26]. Inositol plays a key role in insulin transduction signaling, and the deregulation of inositol metabolism has been demonstrated in different conditions of insulin resistance, which has been associated with several cancers [27,28]. Insulin resistance is involved in the pathogenesis of many cancers, and it is usually due to obesity, which can cause a chronic low-grade inflammation state that can favor neoplastic transformation [29]. Insulin resistance stimulates the hepatic synthesis of insulin-growth factor 1 (IGF-1) and increases its bioactivity by reducing insulin-growth factor binding protein 1 (IGFBP-1) and 2 (IGFBP-2) levels [30]. IGF proteins can likely promote mitogenic activation by binding not only their own receptors, but also the insulin receptor, which shares 84% of its identity with the IGF-1 receptor [31]. Functional activation of both the insulin receptor and the IGF-1 receptor results in the activation of mitogenic-activated protein (MAP) kinase and the PI3K/Akt pathway, frequently observed in malignant cells [27]. 

The PI3K/Akt pathways represent a key step in the activation of survival pathways, including Wnt and NF-kB activation [32], cell cycle progression and cell growth [33]. In this scenario, inositol may directly affect the carcinogenesis process by modulating several critical processes downstream of insulin stimulation, including endocrine modulation, antioxidant defenses and oxidative glucose metabolism [27]. Inositol and some of its phosphate derivatives (such as InsP6) have been demonstrated to reduce PI3K expression and Akt activation in neoplastic cells by inhibiting Akt phosphorylation [16]. The role of inositol in PI3K blocking could even go as far as reducing the aggressiveness of tumor cells, since PI3K/Akt pathway activity is required for triggering the epithelial–mesenchymal transition [15]. It has been demonstrated that inositol displays multi-targeted effects on several biochemical pathways involved in the epithelial–mesenchymal transition (EMT), a physiological process in which epithelial cells acquire the motility and the invasiveness of mesenchymal cells, leading to the promotion of the metastatic process. In breast cancer cells, inositol has been demonstrated to induce an EMT reversion through a significant downregulation of PI3K/Akt activity [15]. This evidence is supported by another *in vitro* study demonstrating that InsP6 blocks epidermal growth factor-induced PI3K activity in a dose-dependent manner in JB6 cells. Further strong evidence of the anti-tumoral effect of InsP6 in blocking the activation of PI3K derives from the impairment of epidermal growth factor or phorbol ester-induced JB6 cell transformation and extracellular signal-regulated protein kinase activation, as well as activator protein 1 activation [16]. 

**Figure 2:**
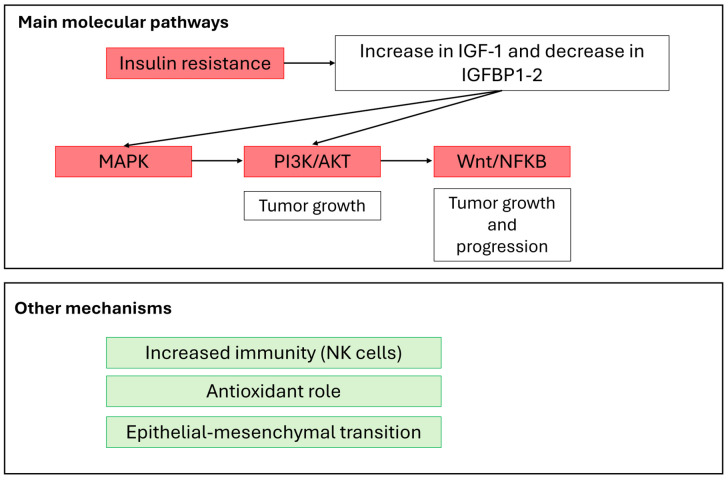
Effects of inositol on mechanisms of neoplastic development and progression. Inositol is able to inhibit the MAPK, PI3K/AKT and Wnt/NFKB pathways, both directly and through a reduction in insulin resistance. Pathways reported in red are blocked by inositol. In addition, inositol enhances immunity, reduces oxidative stress and the epithelial–mesenchymal transition. Abbreviations: IGF-1: insulin growth factor-1; IGFBP: IGF binding protein; MAPK: mitogen-activated kinase; PI3K: phosphoinositide-3 kinase; NFKB: nuclear factor kappa-light-chain-enhancer of activated B cells; NK: natural killer; Wnt: Wingless-related integration site.

Several epidemiological data have already shown an association between the use of metformin, an insulin-sensitizing drug, and a lower incidence of thyroid cancer and pancreatic neuroendocrine tumors (pNETS) in diabetic patients. Considering the proposed anticancer mechanism, an indirect modulation targeting insulin resistance should definitely be considered [34]. Figure 2 reports a summary of the main mechanisms proposed for inositol’s role as a blocker of carcinogenesis and tumor development.

### 3.2. Inositols and Thyroid Cancer

Among endocrine tumors, thyroid cancer is undoubtedly the most common, with an increasing incidence in recent decades [35]. Palpable nodules constitute the smallest proportion of thyroid nodules (approximately 5%), but ultrasound is able to detect even the smallest thyroid nodules, with higher frequencies in women and the elderly. Most diagnosed nodules are benign and asymptomatic, requiring only observation and follow-up. Progression to thyroid cancer occurs in 7–15% of nodules and several risk factors, such as family history, age, sex [36,37] radiation exposure [38], can influence this process.

Different ultrasonographic features, including elastosonographic examination, allow us to estimate the risk of thyroid nodule malignancy. Poor elasticity due to alterations in the mechanical properties of the thyroid tissue is associated with an increased risk of malignancy [39]. In indeterminate lesions (TIR3A thyroid nodules), high elasticity has been shown to predict benignity, having been found in 102 out of 111 benign nodules and in only 1 out of 31 carcinomas (*p* < 0.0001) [40]. Nordio et al. demonstrated that supplementation with myo-Ins, with the addition of selenium, induces a morphological change in thyroid nodules classified as class I or II (at low and intermediate risk according to ultrasound classification of the AACE/ACE/AME 2016 guidelines [41]), reducing their size and stiffness, as evaluated by elastosonography [42]. The effect of myo-Ins supplementation on thyroid nodules can be traced back to the relevant role of phosphoinositides in thyroid function and their actions on pathways involved in cell proliferation.

Myo-Ins is a precursor of phosphoinositides, and among these, phosphatidylinositol 4,5-bisphosphate (PIP2) is the precursor of IP3, the second messenger of different hormones, including thyroid-stimulating hormone (TSH). As a second messenger in the phospholipase C (PLC)-dependent inositol phosphate Ca^++^/DAG pathway, myo-Ins contributes to thyroid hormone synthesis through H_2_O_2_ production.

Subsequently, it is natural to think that the depletion of inositol or the impairment of its signaling pathway linked to TSH may lead to a state of hypothyroidism. Several clinical studies have demonstrated that treatment with myo-Ins plus selenium is able to significantly reduce TSH levels in patients with subclinical hypothyroidism with or without autoimmune thyroiditis and that a reduction in TSH is also associated with a decline in anti-thyroid antibodies [43]. A systematic review including 28 studies with a total of 42,032 subjects and 5786 thyroid cancer cases demonstrated that higher serum TSH concentrations are associated with an increased risk of thyroid cancer, with a probable key role of anti-thyroid antibodies [44]. A meta-analysis including 53,538 participants demonstrated that each 1 mU/L increase in serum TSH was associated with a 14% greater risk of thyroid cancer for all histological thyroid cancer, 16% for differentiated thyroid carcinoma and 22% for papillary thyroid carcinoma (PTC), but, conversely, high serum TSH was associated with a reduced risk of follicular thyroid carcinoma (FTC) [45].

The relationship between thyroid autoantibodies and thyroid cancer is still a matter of debate [46,47,48]. Other studies have not found statistical significance; however, the risk of developing thyroid papillary thyroid carcinoma in Hashimoto thyroiditis patients might be considered [49]. Supplementation with myo-Ins and selenium has been shown to reduce both thyroid antibody titer and TSH levels [50,51]; consequently, a reduction in TSH levels, a risk factor for thyroid cancer, could be considered a protective factor against the development of malignant thyroid neoplasms. 

Myo-Ins could be considered a thyroid cancer oncometabolite [52]. Proton nuclear magnetic resonance spectroscopy-based metabolomics demonstrates the ability to differentiate tumors from benign nodules. This is particularly interesting in the case of “indeterminate” nodules; metabolomics has demonstrated increased levels of phenylalanine, taurine and lactate, and reduced levels of choline/choline derivatives, myo- and scyllo-inositol, in malignant nodules compared to benign ones, suggesting a possible depletion of inositols in the case of malignant cells [53].

A crucial and interesting finding is the increased activity of the PI3K/Akt pathway observed in thyroid cancers. Ringel et al. demonstrated that human thyroid cancer tissues, especially FTC, are characterized by an increased expression of Akt1 and Akt2 and increased Akt activity compared with normal thyroid tissue [54]. Both in FTC and anaplastic thyroid cancers (ATCs), mutations or amplifications of the PIK3 synthesizing enzyme *PI3KCA* gene have been reported [55,56,57]. PIK3CA amplification was associated with an overexpression of the PIK3CA protein and phosphorylation of Akt, suggesting that this alteration represents a relevant oncogenic event in thyroid tumorigenesis via the PI3K/Akt pathway [57,58]. In particular, PIK3CA amplifications could exert a promoting role in the initial stages of thyroid tumorigenesis, since they are also found in benign thyroid adenoma (BTA), although with low prevalence [57]. In addition, other classical fusions associated with differentiated thyroid cancer, such as rearrangement involving the *RET* gene (RET/PTC), appear to activate the PI3K/AKT pathway [59,60]. Furthermore, *RAS* mutations, identified in many thyroid tumors, particularly in FTC and in the follicular variant of papillary thyroid carcinoma (FV-PTC) [57,61], may enhance thyroid tumorigenesis through their interaction with the PI3K/Akt pathway, which is a common and early event in FTC [62]. 

The role of PI3K signaling has also been detected in thyroid cancer progression. Studies in mouse models with metastatic FTC have demonstrated enhanced AKT activation both in primary and metastatic tumor tissue [63]. Vasko et al. reported that activation of AKT, particularly AKT 1, is associated with tumor invasion and metastasis both in FTC and PTC [64]. The investigation of molecular pathways in thyroid nodules caught the attention of some researchers who investigated the immunohistochemical expression of HGF, c-met, STAT3, phosphorylated-STAT3 (pSTAT3), PI3K, Akt and Rho in 83 benign thyroid nodules and 46 thyroid cancers (20 PTCs, 16 FTCs and 6 ATCs). The study showed that all seven proteins were expressed in 15% of follicular adenomas and all PTCs expressed the combination hepatocyte growth factor (HGF)/cmet/STAT3/pSTAT3/PI3. Among FTCs, the proportion of PI3K+ cells correlated with both the clinical phenotype and pathological stages [65].

In thyroid cells, the proliferative pathway PI3K can be activated by both TSH receptor (TSH-R) and tyrosine kinase receptors, such as insulin receptor (IR) and insulin-like growth factor 1 receptor (IGF-1R). In this context, TSH exerts its proliferative action on thyrocytes both through its own receptor and by inducing IGF-1R production and potentiating the IGF1-mediated proliferation pathways associated with PI3K and mitogen-activated protein kinase (MAPK) [66]. Thus, TSH and IGF1 are synergistic in promoting thyrocyte proliferation [67]. IGF1 is mainly produced locally in either a paracrine or autocrine manner and although it is present both in normal and in thyroid cancer tissues, IGF1 and its receptor IGF-1R are found to be overexpressed in thyroid cancer cell lines. 

Considering the effects of insulin on promoting cell growth and the epidemiological evidence of the high prevalence of insulin resistance in patients with DTC [68], it may be possible to assimilate the effect of inositol to that of metformin, studied in goiter thyroid cells and in thyroid carcinoma cell lines [69]. Metformin has been demonstrated to reduce the cell viability of thyroid carcinoma cells, blocking cell cycle progression and inducing apoptosis, reducing the insulin-stimulated cell proliferation of thyroid carcinoma cells and their cancer stem cells.

**Figure 3:**
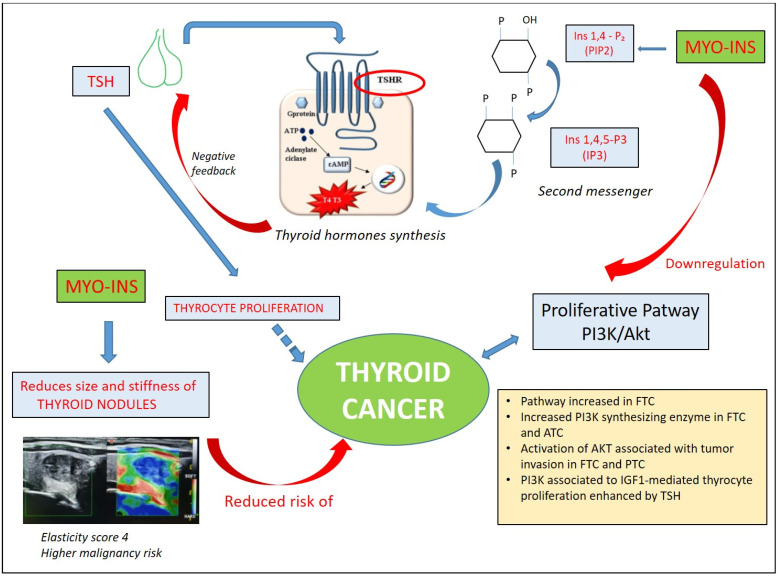
Effect of inositol on thyrocyte proliferation and thyroid cancer: myo-Ins, through its phosphoinositide derivatives (PIP2 and PI3) as the second messenger of TSH, contributes to the synthesis of thyroid hormones. This leads to a reduction in TSH levels, the hormone involved in the proliferation of thyrocytes and an increased risk of developing potentially malignant thyroid nodules. Myo-Ins regulates the PI3K/Akt pathway, which is increased in several types of thyroid cancer: FTC, ATC and PTC Abbreviations: FTC: follicular thyroid cancer; ATC: anaplastic thyroid cancer; PTC: papillary thyroid cancer; IGF1: insulin-like growth factor.

The described effect of myo-Ins and IP6 in anticancer prevention, by inhibiting PI3K and the downregulation of Akt activity [15,16] and reducing insulin resistance, proposes myo-Ins as a protective factor for the malignant transformation of thyroid nodules and metastatic spread development (Figure 3). Some studies on thyroid nodules demonstrated the efficacy of myo-Ins and selenium in nodule proliferation, but the potential role of this supplementation should be explored further. 

### 3.3. Inositols and Adrenal Tumors

Adrenal tumors include tumors arising from cortical (adrenocortical adenomas/carcinomas) and medullary tissue (pheochromocytomas), and both of them can be either benign or malignant. Pheochromocytomas and paragangliomas (PPGLs) derive from the chromaffin cell of the neural crest (from the adrenal medulla and sympathetic paraganglia, respectively); therefore, they should be considered neuroendocrine tumors. Although the majority of PPGLs are benign tumors, up to 20% of them can be malignant [70]. Based on genomic studies, PPGL tumors can be grouped into three clusters. Cluster 1 includes tumors derived by mutations in *SHDx*, *VHL*, *EPAS1*, *FH* and MDH2; cluster 2 comprises tumors with underlying *RET*, *NF1*, *TMEM127*, *HRAS*, *KIF1B* and *MAX* mutations; and the third cluster, called the Wnt-altered subtype, is derived from *MAML3* fusions and *CSDE1* somatic mutations [71,72,73]. 

Using mass spectrometry in 344 PPGLs, Murakami et al. showed that compounds related to inositol metabolism were decreased in cluster 1 and inositol cyclic phosphate, as an inositol-related metabolite, was also reduced in SHDH- and VHL- knockdown PC12 cells [74], thus suggesting a possible target therapy in patients with those specific mutations. Furthermore, several studies have suggested that insulin secretion is impaired in patients affected by PPGL, likely as a result of the inhibitory effect of catecholamines on pancreatic β cells; consequently, catecholamines antagonize insulin action in target organs, triggering an insulin resistance status [75,76]. Endogenous catecholamine excess in patients affected by pheochromocytoma can induce or aggravate insulin resistance both in patients with type 2 diabetes and in patients with normal glucose tolerance [77]. 

In the context of the PI3K/Akt pathway, in which inositol seems to play a key role, Fassnacht et al. observed an increased expression of Akt both in pheochromocytomas and adrenocortical carcinomas compared to normal adrenals or adenomas. Furthermore, the activated form, p-AKT, has also been reported to be overexpressed in pheochromocytomas [78]. Adler et al. demonstrated that pharmacologic inhibition of the PI3K-Akt pathway in pheochromocytoma cells downregulated the neuroendocrine phenotype, decreased hormonal secretion and promoted apoptosis [79].

Fewer data exist regarding the expression of p-AKT in adrenocortical carcinomas. The mammalian target of rapamycin (mTOR), a kinase of the PI3K-Akt signaling pathway, is a crucial intracellular mediator of the activity of growth factors receptors, comprising vascular endothelial growth factor (VEGF) and IGFs. Dysregulation of the mTOR pathway is a known feature of many tumors; therefore, the mTOR pathway is considered a target for antineoplastic therapy in different tumors. The role and functions of mTOR and its signaling pathway in the normal and tumoral adrenal gland have not been completely clarified but a dysregulation of AKT, leading to growth-simulating effects, has been described in adrenocortical carcinomas and pheochromocytomas [80].

It is worth highlighting the key role of insulin resistance in adrenocortical tumor growth [81], even if, at present, there is no unequivocal evidence of a clear causal relationship between hyperinsulinemia and adrenal tumors. Benign non-functioning adenomas are commonly associated with insulin resistance [76], although it is still a matter of debate whether this association is mediated by undetected cortisol secretion from adrenal tumor or if insulin resistance and the compensatory hyperinsulinemia stimulate adrenal tumor cell growth [82]. Additionally, in malignant neoplasms, hyperinsulinemia may potentiate the proliferative effect of IGF-2, which is overexpressed in 90% of adrenocortical carcinomas [83,84], through the binding of insulin receptor A (IR-A), IGF-1 receptor (IGF-1R) and hybrid receptor IR-A/IGF-1R. The binding of insulin or IGF-2 to these receptors leads to the activation of downstream PI3K/AKt pathways [85].

**Figure 4:**
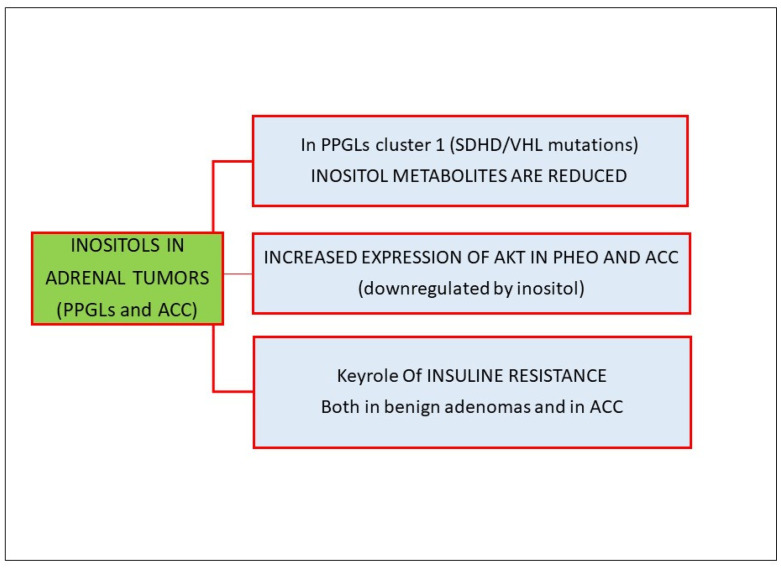
The role of inositols in adrenal tumors: in medullary tissue-derived tumors, particularly in cluster 1 tumors, inositol-related metabolites are decreased. Both in PHEO and in ACC, the Akt pathway is overexpressed. Insulin resistance plays a key role both in benign adrenal neoplasms and in ACC, where hyperinsulinemia may enhance the proliferative effect of IGF-2, which is overexpressed in 90% of ACCs. Abbreviations: PPGLs: pheochromocytomas/paragangliomas; PHEO: pheochromocytoma; ACC: adrenocortical carcinoma.

Figure 4 summarizes the role of inositol in adrenal tumors. 

### 3.4. Inositols and Pituitary Neuroendocrine Tumors (Pit-NETs)

Low evidence is available regarding the role of inositol in Pit-NETs. Jones et al. demonstrated different expression levels of phosphoinositide in 29 pituitary adenoma tissues, which were divided into two groups, one with a high basal turnover and another with low basal phosphoinositide. The first group was associated with kinine response and produced interleukin-6, which is involved in promoting mitogenic growth and pituitary tumorigenesis, suggesting a possible role of inositols in the control of mechanisms involved in the cell growth of some, but not all, pituitary tumors [86].

A more recent systematic review investigated the metabolomic aspects of pituitary adenomas using mass spectrometry, suggesting an alteration of amino acid metabolism in these tumors. In detail, the authors demonstrated that phosphethanolamine, N-acetyl aspartate and myo-Ins are downregulated in prolactinomas, hypothesizing that a possible deficiency of these biomolecules in some tumors could determine their pathogenesis [87]. 

Currently, there are limited studies suggesting the growth-inhibitory effects of metformin, an anti-diabetic and insulin-sensitizing drug, on PitNETs. The antiproliferative effects of metformin have been observed on an ACTH-secreting mouse corticotroph tumor cell line and growth hormone-secreting PitNET cell lines, GH3 and GH1 [88]. In ACTH-secreting AtT20 cells, metformin inhibited cell proliferation by activating the AMPK signaling pathway and inhibiting the IGF-1R/AKT/mTOR pathway. In a recent study performed on primary cell cultures derived from PitNETs, metformin treatment inhibited cell viability in ACTH-secreting adenomas and non-functioning pituitary adenomas, but not prolactinomas, and in GH-secreting adenomas [89]. Inositol, which shares the same Akt/mTOR signaling pathway, could be hypothesized as a possible concomitant influencing factor in the cellular growth of Pit-NETs.

### 3.5. Inositols and Neuroendocrine Neoplasms

Neuroendocrine neoplasms (NENs) arise from neuroendocrine cells, located in all parts of the body, though the most common sites of origin are the lung and the gastro-entero-pancreatic (GEP) system. The incidence and prevalence of these tumors, while rare, is increasing [90]. The term NENs includes the well-differentiated forms, also known as neuroendocrine tumors (NETs), which have a low proliferation rate and are slow-growing, and the poorly differentiated neoplasms, also known as Neuroendocrine Carcinomas (NECs), characterized by a high proliferation rate and clinical aggressiveness [91]. NENs can be non-functioning or functioning, with the latter characterized by the secretion of hormones and peptides associated with a specific syndrome [92]. 

Many research articles demonstrated a pathogenetic role of the PI3K/Akt/mTOR pathways in the development of NENs. mTOR and PI3K signaling can regulate cell growth, proliferation, migration, survival and angiogenesis [93]. 

The *PIK3CA* gene, encoding for the catalytic subunit of class I PI3K, can be mutated in a sub-group of pNENs [94]. Preclinical studies using PI3K inhibitors (LY294002) in rat-derived GEP-NEN cell lines result in the inhibition of VEGF secretion by neoplastic endocrine cells [95]. Similarly, a study on BON cells, one of the few neuroendocrine cell lines available, has demonstrated that LY294002 blocks the constitutive activation of PI3K and ERKs [96]. PI3K signaling is also able to reduce neuroendocrine secretion in BON and QGP-1 cells [97]. 

mTOR hyperactivation has frequently been detected in neuroendocrine tumors [98,99] but also in poorly differentiated NECs [100]. Mutations of genes of the mTOR pathway, such as *PTEN*, *TSC2* and *PIK3CA*, have been detected in about 16% of sporadic pancreatic NETs [101]. Additionally, low expression of *TSC2* and *PTEN* was associated with reduced disease-free and overall survival in pancreatic NENs [102]. Everolimus, a mTOR inhibitor, is currently approved for the treatment of GEP and lung NETs [103]. However, NENs can develop resistance to everolimus, and one of the proposed pathways is the activation of PI3K, which could be potentially overcome by the use of inositols.

Finally, NEN cells are able to produce high amounts of IGF 1. In gastrinomas, high levels of IGF-1 and its receptor (IGF-1R) were associated with tumor development, progression and aggressiveness [104]. 

To date, there are no data on inositol use in patients affected by neuroendocrine cells, but studies on other cancer cells have suggested a role of IP6 in the induction of cell apoptosis by inhibiting the Akt/mTOR pathway and PI3k pathways, supporting the rationale for the use of inositols in combination with everolimus in NENs.

## 4. Clinical Use of Inositols and New Therapeutic Perspectives

Inositol supplementation (in the form of myo-Ins and D-chiro-inositol) is currently used as an effective alternative to the classic insulin-sensitizing molecule, metformin, in many gynecological and endocrinological diseases, such as PCOS, gestational diabetes mellitus (GDM) and male infertility [26].

In a recent systematic review and meta-analysis of 36 randomized controlled clinical trials with a total of 1691 PCOS patients randomized to inositol, metformin or placebo, it was demonstrated that inositol was superior to placebo and not inferior to metformin in normalizing menstrual cycles and superior to placebo in reducing BMI, blood levels of testosterone, androstenedione glucose and AUC insulin [105].

Regarding the use of inositol in the treatment of endocrine tumors, despite the promising evidence described above, we are still far from a possible clinical use. In many other tumor histotypes, there are already robust *in vitro* and *in vivo* studies that reinforce their potential use in human tumors. In a small pilot clinical trial involving 22 patients suffering from metastatic colon cancer, the combination of IP6 and inositol (InoCell) as an adjuvant to conventional chemotherapy caused a reduction in tumor growth, also lowering the side effects of chemotherapy (leukopenia, thrombocytopenia, nausea and vomiting) [106,107].

## 5. Conclusions

This review highlights the role of inositols in the pathogenesis and progression of the main types of endocrine and neuroendocrine cancers, proposing a solid rationale for the use of inositol supplementation as an adjuvant treatment in this field of oncology. 

We believe that the current evidence on the molecular mechanisms of carcinogenesis, particularly in thyroid and adrenal tumors, can support researchers’ decisions to apply inositol in *in vitro* and *in vivo* studies in these settings to confirm these hypotheses. 

## Abbreviations

myo-Ins	myo-inositol
IP6	inositol hexakisphosphate
IP3	inositol triphosphate
IGF-1	insulin-growth factor 1
IGFBP-1	insulin-growth factor binding protein 1
IGFBP-2	insulin-growth factor binding protein 2
MAP	mitogenic-activated protein
PI3K/Akt	phosphoinositide-3 kinase/Akt
EMT	epithelial–mesenchymal transition
pNETS	pancreatic neuroendocrine tumors
PIP2	phosphatidylinositol 4,5-bisphosphate
TSH	thyroid-stimulating hormone
PLC	phospholipase C
PTC	papillary thyroid carcinoma
FTC	follicular thyroid carcinoma
BTA	benign thyroid adenoma
FV-PTC	follicular variant of papillary thyroid carcinoma
pSTAT3	phosphorylated-STAT3
HGF	hepatocyte growth factor
MAPK	mitogen-activated protein kinase
PPGL	paragangliomas
mTOR	mammalian target of rapamycin
VEGF	vascular endothelial growth factor
Pit-NET	pituitary neuroendocrine tumors
NEN	neuroendocrine neoplasm
NET	neuroendocrine tumor
NEC	neuroendocrine carcinoma
GEP	gastro-entero-pancreatic system
PHEO	pheochromocytoma
ACC	adrenocortical carcinoma
PCOS	polycystic ovary syndrome
GDM	gestational diabetes mellitus
